# Mechanism of miRNAs and miRNA-mRNA Regulatory Networks in Modulating Drug Resistance in HER2-Positive Breast Cancer: An Integrative Bioinformatics Approach

**DOI:** 10.3390/cancers16233962

**Published:** 2024-11-26

**Authors:** Thanh Hoa Vo, Edel A. McNeela, Orla O’Donovan, Sweta Rani, Jai Prakash Mehta

**Affiliations:** 1 Pharmaceutical and Molecular Biotechnology Research Center (PMBRC), Department of Science, South East Technological University, X91 K0EK Waterford, Ireland; thanhhoa.vo@postgrad.wit.ie (T.H.V.); edel.mcneela@setu.ie (E.A.M.); orla.odonovan@setu.ie (O.O.); sweta.rani@setu.ie (S.R.); 2 Department of Applied Science, South East Technological University, R93 V960 Carlow, Ireland

**Keywords:** HER2-positive breast cancer, miRNAs, treatment response, HER2 targeted therapy, drug resistance, bioinformatics

## Abstract

Background: HER2-positive breast cancer is an aggressive subtype where innate/acquired resistance to targeted drugs remains a challenge. This study aims to uncover the underlying mechanisms of HER2 drug resistance through miRNA analysis and target identification. Methods: MiRNA datasets were systematically retrieved from the GEO database, and differential expression analysis was conducted for both miRNA and mRNA datasets. Functional analyses were also conducted to validate the identified miRNAs and assess their clinical relevance. Results: We identified 113 differentially expressed miRNAs (DEMs) and 923 target genes. Validation was performed using external mRNA datasets, and intersection with significant genes identified 110 overlapping genes associated with HER2 drug resistance. Further analyses included functional enrichment, construction of a protein–protein interaction (PPI) network, identification of key hub genes such as BCL2, FOS, and CXCR4, and assessment of clinical relevance through survival analysis and immunohistochemistry (IHC) assessments. Conclusions: This integrative approach unveils a complex landscape of HER2 drug resistance in breast cancer, identifying crucial miRNAs, target genes, and significant pathways. The findings offer novel insights into the mechanisms governing drug resistance and highlight the potential for enhancing therapeutic strategies. Future studies are necessary for experimental validation to further explore the complex mechanisms involved.

## Simple Summary

HER2-positive breast cancer is the most aggressive form of cancer, accounting for 20–25% of all breast cancers, with poor overall survival. HER-targeted drugs (trastuzumab, lapatinib, and pertuzumab) have improved patient outcomes, but innate/primary and acquired resistance present substantial clinical challenges. Growing tumors often secrete small RNA sequences (miRNA) that are stable in the blood, and detecting them can contribute to enhanced treatment plans and reverse resistance. This study aims to investigate the underlying mechanisms of HER2 drug resistance through miRNA analysis and target identification. The results from this study contribute to better treatment of HER2-breast cancer resistance to targeted therapies.

## 1. Introduction

Breast cancer is a common lethal cancer in women. A recent report (2010–2019) from the American Cancer Society revealed a yearly increase of 0.5% in the rate of female breast cancer incidence [1]. The human epidermal growth factor receptor 2 (HER2)-positive subtype, which accounts for 20–25% of all breast cancer cases [2,3], has gained substantial attention owing to its aggressive nature and resistance to treatment. This highly aggressive neoplasm is characterized by HER2-mediated activation of oncogenic pathways and is poorly responsive to cytotoxic chemotherapy [4].

HER2-targeted therapies [5,6,7,8,9,10,11], which were designed to target the HER2 receptor, have achieved significant improvement in managing this subtype. Despite the initial promise of HER2-targeted therapies, therapeutic resistance presents a daunting challenge, limiting the effectiveness of these treatments. This resistance significantly impairs patient prognosis and hampers progression-free survival and overall survival rates [12,13]. This issue necessitates the need for new treatment strategies with potential targets to overcome drug resistance.

MiRNAs are small non-coding RNAs known to modulate gene expression at the post-transcriptional level and regulate many biological processes, including carcinogenesis [14,15]. Recent research links miRNAs with the development of drug resistance in breast cancer. In particular, the abnormal expression of miRNAs has been investigated for its role in causing anti-HER2 therapeutic resistance [16,17]. miRNAs influence resistance to HER2-targeted therapies in breast cancer by regulating key genes involved in pathways critical for cancer cell survival and proliferation [18]. For instance, miR-21 drives trastuzumab and chemotherapy resistance by targeting *PTEN* and *PDCD4*, thereby activating the *PI3K* pathway and supporting processes like epithelial-to-mesenchymal transition (EMT), which can sustain resistance and invasiveness [19]. Similarly, miR-221 promotes resistance through the *PI3K/AKT* pathway by downregulating *PTEN*, further enhancing cancer cell proliferation and survival independently of immune signaling [20]. Further exploration of miRNA-driven mechanisms underlying HER2 drug resistance and potential biomarkers of drug response can help provide effective options for treatment prognosis and personalized medicine.

Recent advancements in technologies and high-throughput datasets have catalyzed the growth of bioinformatics. Computational tools have been intensively exploited to dissect the role of miRNA in the complex molecular network in cancer [21,22,23]. It is a common practice to use microarray analysis of gene expression profiles to identify cancer-related genes and pathways. With the current availability of the public database [24,25,26], breast cancer datasets can easily be retrieved and analyzed. However, the majority of these studies have been primarily focusing on the identification of gene biomarkers, understanding cancer subtypes, or exploring chemotherapy resistance mechanisms in breast cancer [27,28,29]. Despite the potential significance of miRNA biomarkers in drug resistance, limited studies have focused on studying miRNA biomarkers in the context of targeted therapy resistance in HER2+ breast cancer. This underscores the need to explore miRNAs regulating drug resistance in this aggressive breast cancer subtype to improve therapeutic strategies and enhance treatment outcomes.

In this study, we identified DEMs associated with breast cancer targeted drug resistance using systematic meta-analysis of the miRNA datasets and predicted their target genes. We validated the target genes with three external mRNA datasets. All datasets were retrieved from the Gene Expression Omnibus (GEO). Preprocessing and normalization were performed before further analysis. The significantly differentially expressed genes (DEGs) associated with HER2 drug resistance in breast cancer were used for further functional and pathway enrichment analysis. Moreover, a protein–protein interaction (PPI) network was constructed. Furthermore, immunohistochemistry and survival analysis were employed to evaluate key hub genes.

## 2. Materials and Methods

### 2.1. Data Acquisition and Preprocessing

In this study, both RNA-sequencing and microarray gene expression profiles were sourced from the Gene Expression Omnibus (GEO) database (https://www.ncbi.nlm.nih.gov/geo/ accessed 22 May 2023) [25], focusing specifically on datasets related to drug resistance in HER2+ breast cancer. Systematic extraction and retrieval of miRNA datasets were conducted using the GEOquery package in R. We also conducted exploratory analyses to assess sample quality and identify any potential outliers from miRNA datasets. Results of these exploratory analyses can be found in Supplementary Figure S2. Three miRNA datasets (GSE47011, GSE197822, and GSE101841) available as of May 2023 and three validation mRNA datasets (GSE132055, GSE89216, and GSE121105) were utilized for our bioinformatics analysis. Table 1 details the information on the retrieved datasets. Substantial missing values were discovered in the GSE47011 expression data, and these were imputed using the MissForest algorithm, an iterated random forest method [30]. All miRNA datasets were then log_2_-transformed, a necessary step to ensure comparability across datasets and reduce the impact of high-magnitude changes, making the data more symmetric. These transformed datasets were subsequently used for expression analysis and microarray meta-analysis.

### 2.2. Differential Expression Analysis and Meta-Analysis

Differential expression analyses were performed on the two groups of samples, resistance and control, in each Gene Expression Omnibus (GEO) dataset, a public functional genomics data repository. Differentially expressed microRNAs (DEMs) were identified using the Linear Models for Microarray and RNA-Seq Data (LIMMA) R/Bioconductor package [31] and DEGs were identified using the GEO2R tool, an interactive web application in the GEO database that leverages the LIMMA package to compare different groups of samples. It allows for the identification of genes differentially expressed across experimental conditions, specifically resistant and control groups in this study. Each gene’s expression was calculated based on the false discovery rate using the Benjamini–Hochberg method (FDR, *p* < 0.05). Besides the adjusted *p*-value < 0.05 that has been corrected for multiple comparisons, a fold change cutoff of log-fold change logFC > 1 was also used as the primary criterion to interpret DEG results.

The combined effect size (ES) was used in the microarray meta-analysis of the miRNA datasets. This metric, expressed as the difference between two group means divided by the standard deviation, enables the comparison of findings across studies. In this context, the group means are represented by the logFC of miRNA expression between the two groups: resistance and control.

Variations in logFC values are expected since different experimental conditions and techniques were used for each dataset. To account for the precision of these logFC estimates, we calculated the standard error (StdErr) for each dataset by dividing the logFC by the corresponding t-value, based on the statistical relationship between logFC, StdErr, and t-value. Calculating StdErr for each dataset allowed us to weigh the effect sizes according to their precision in the meta-analysis. For the meta-analysis, we applied a random-effect model, which accommodates inter-study variability and provides a combined effect size across datasets.

To identify the appropriate model for meta-analysis, we initially assessed the presence of heterogeneity among the studies using Cochran’s Q test [32]. This test is used to determine whether the true effect sizes are homogeneous across different studies. A significant result (*p* < 0.05) in the Q test indicates heterogeneity, leading us to choose a random-effect model for our meta-analysis. This model accounts for variations between studies and is widely accepted in meta-analysis.

Each miRNA was individually assessed, and the resulting effect sizes and *p*-values were adjusted for multiple comparisons using the Benjamini–Hochberg method. MiRNAs with an adjusted *p*-value of less than 0.05 were considered significantly differentially expressed. A set of DEMs was thus obtained.

### 2.3. Prediction of Target Genes for DEMs and Identification of Significant Drug-Resistance-Associated DEGs

Upon identifying DEMs, this set was further refined to include only those pertaining to humans, as indicated by the prefix “hsa” in their identifiers. The resulting set was used for subsequent target prediction analysis.

To predict miRNA targets, we utilized the ‘multiMiR’ R package [33]. This package integrates information from 14 databases, providing both experimentally validated and computationally predicted target interactions between miRNAs and mRNAs, along with their disease and drug associations. For each significant miRNA, validated miRNA-mRNA interactions from these databases were retrieved. The resulting list of target mRNAs for the DEMs was saved for subsequent analyses.

Subsequently, we identified significant genes associated with drug resistance in HER2+ breast cancer. This was achieved by intersecting the significant DEGs identified in the external validation mRNA datasets with the list of DEM target genes.

### 2.4. Functional Enrichment Analysis and Protein–Protein Interaction (PPI) Network Construction

Functional annotation and enrichment analysis were conducted with Gene Ontology (GO) [34] and Kyoto Encyclopedia of Genes and Genomes (KEGG) pathways [26], using the ClueGo plugin [35] (version 2.5.10) in Cytoscape [36] (version 3.10.0), and the EnrichR tool [37]. These analyses helped in examining the associated biological processes, molecular functions, cellular components, and signaling pathways relevant to significant DEGs. In the GO analysis, the significance of the enrichments was determined using the Bonferroni method with a kappa score of 0.96 and a cutoff value of *p* < 0.05. For KEGG enrichment analysis, enriched pathways were identified with an adjusted *p*-value of less than the 0.05 cutoff. Visualization of the results was performed using Cytoscape. Additionally, to explore potential functional relationships among the proteins encoded by the target mRNAs, a protein–protein interaction (PPI) network was constructed using the STRING database [38].

### 2.5. Validation and Prognostic Evaluation

**Figure 1:**
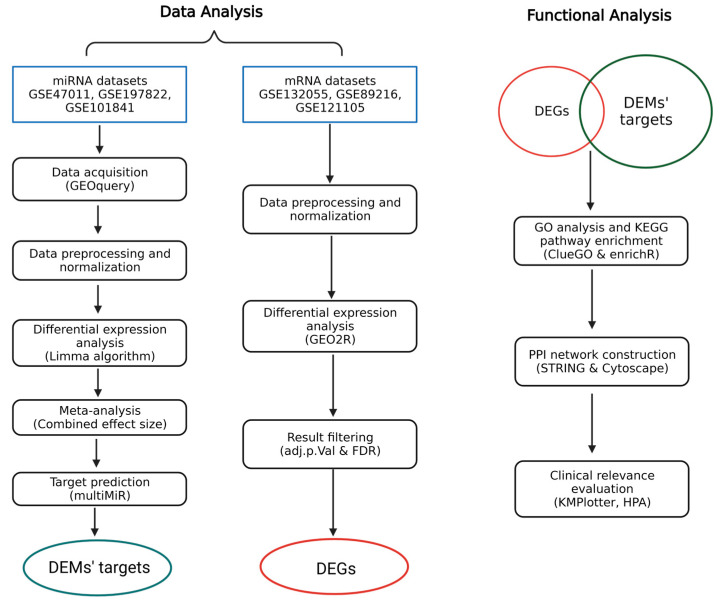
Schematic of our study.

The biological and clinical relevance of the identified significant genes were further validated to assess their impact on patient outcomes. To evaluate the impact of these genes on overall survival, we utilized KMPlotter [39], a tool that integrates survival data from multiple studies and enables Kaplan–Meier survival analysis. Additionally, the protein expression profiles of the significant genes were explored using immunohistochemistry data available in the Human Protein Atlas [40]. This approach allowed both the genetic and protein-level implications of the significant genes to be thoroughly assessed, reinforcing the validity of our findings. Figure 1 illustrates the schematic method of our study.

## 3. Results

### 3.1. Data Selection

We found 64 records from the systematic search of the GEO database up to May 2023 under specific keywords. Based on stringent inclusion criteria, including targeted drug resistance in HER2+ breast cancer, miRNA expression data availability, and limited to human studies, 61 datasets were excluded, leaving us with three miRNA datasets (GSE47011, GSE197822, and GSE101841) for further analysis. In each dataset, HER2-targeted drug resistance samples were compared with the control. The data selection process of the study is illustrated by a PRISMA diagram in Supplementary Figure S1.

### 3.2. Identification of DEMs and DEGs

**Figure 2:**
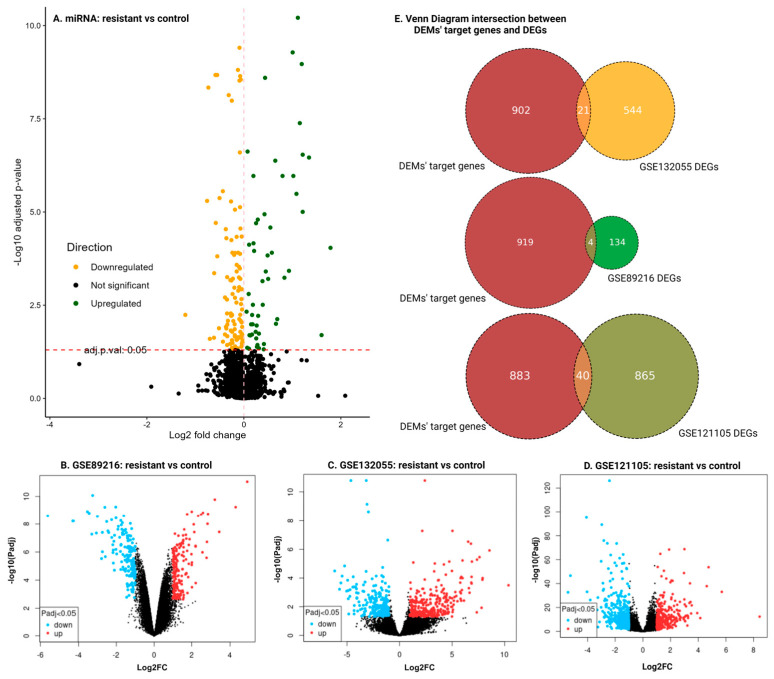
Summary of DEMs and DEGs across multiple datasets. (**A**) Volcano plot for miRNA meta-datasets, the result of the meta-analysis. Yellow points indicate downregulated miRNAs, and green points indicate upregulated miRNAs, with an adjusted *p*-value < 0.05 as the significance threshold. (**B**–**D**) Volcano plots for mRNA datasets GSE89216 (**B**), GSE132055 (**C**), and GSE121105 (**D**), showing differential expression analysis. Blue points represent downregulated genes, and red points represent upregulated genes. In all volcano plots, the Log_2_-fold differences are plotted on the horizontal axis, and the −Log10pvalue differences are plotted on the vertical axis. The significance criteria for identifying DEGs are an adjusted *p*-value < 0.05 and an absolute Log_2_-fold change|Log_2_FC| > 1. (**E**) Venn diagram depicting the intersection between DEM target genes and DEGs, highlighting genes associated with HER2-targeted drug resistance.

In our analysis of multiple datasets, we identified key molecular alterations associated with HER2-targeted drug resistance in breast cancer. For miRNAs, the meta-analysis of three miRNA datasets revealed a total of 113 DEMs, including 56 downregulated (yellow points in Figure 2A) and 57 upregulated miRNAs (green points in Figure 2A). The significance threshold was set at an adjusted *p*-value < 0.05.

For mRNAs, differential expression analysis was conducted on three validation datasets. Specifically, the analysis identified 139 DEGs in GSE89216 (Figure 2B), with 67 upregulated (red points) and 72 downregulated genes (blue points). In GSE132055, 565 DEGs were identified, comprising 240 upregulated (red points) and 325 downregulated genes (blue points) (Figure 2C). The additional dataset GSE121105 was also analyzed with 905 DEGs and is depicted in Figure 2D. The significance criteria for identifying DEGs were an adjusted *p*-value < 0.05 and an absolute Log_2_-fold change |Log_2_FC| > 1.

### 3.3. miRNA Target Prediction and Overlap with DEGs

In this study, we utilized the multiMiR package to predict the target genes of all identified DEMs. The predicted target genes of DEMs were then overlapped with the DEG list identified from our mRNA datasets to enhance the biological relevance of the identified targets. This resulted in 110 genes that were not only differentially expressed in our mRNA analysis but also predicted to be targeted by one or more of the DEMs. The Venn diagram in Figure 2E shows the intersection of the DEMs’ target genes and DEGs.

### 3.4. Integration of Enrichment and PPI Analyses

**Figure 3:**
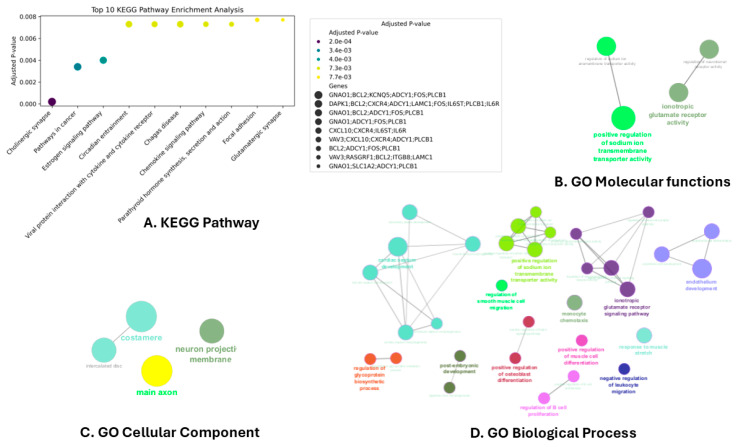
Gene Ontology (GO) and KEGG pathway enrichment analysis for significant overlapped genes associated with HER2-targeted drug resistance. (**A**) KEGG pathway enrichment analysis by EnrichR, where the size of each point represents the number of genes in the term, and color indicates the *p*-value. The analysis highlights key pathways, structures, and mechanisms potentially underlying resistance. (**B**–**D**) GO analyses for molecular functions (**B**), cellular components (**C**), and biological processes (**D**), visualized with ClueGO in Cytoscape. In these plots, color represents different groups, and size corresponds to the number of genes in each term. All terms shown meet a significance threshold of *p* < 0.05.

Enrichment Analysis: We performed GO analysis on the overlapping significant genes associated with HER2-targeted drug resistance to gain insights into the associated biological processes, molecular functions, and cellular components. KEGG pathway enrichment analysis was also performed. Key enriched GO terms and pathways are shown in Figure 3.

The result from GO analysis showed that terms like cardiac septum development and endothelium development are critical terms based on the kappa score. Genes including ankyrin 2 (*ANK2*), heart development protein with EGF-like domains 1 (*HEG1*), roundabout guidance receptor 2 (*ROBO2*), slit guidance ligand 2 (*SLIT2*), T-box transcription factor 1 (*TBX1*), and transforming growth factor beta receptor 3 (*TGFBR3*) were identified within significant nodes with Bonferroni step-down-corrected *p*-values.

**Figure 4:**
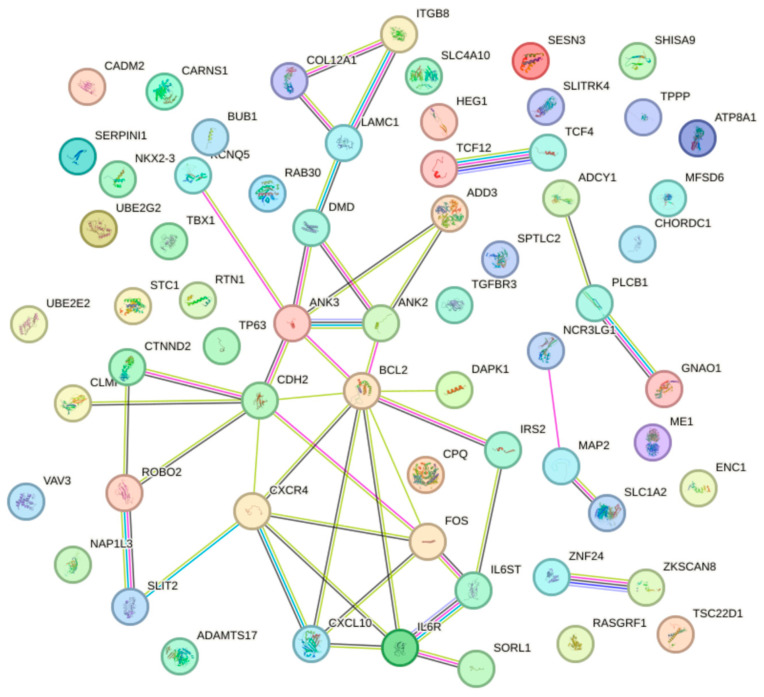
Protein–protein interaction (PPI) network for HER2-targeted drug resistance. This figure represents the PPI network constructed using STRING, showcasing the interconnectivity among significant genes associated with HER2 drug resistance in breast cancer. Key hub genes like BCL2 are depicted, along with their connections to other relevant proteins such as FOS, CXCR4, CXCL10, IL6R, IL6ST, IRS2, DAPK1, and CDH2. Separate clusters involving PLCB1, ADCY1, and GNAO1 are also visualized.

Several pathways were significantly enriched in the KEGG enrichment analysis, including cholinergic synapse, pathways in cancer, and estrogen signaling pathway. Key genes involved in these pathways such as BCL2 apoptosis regulator (*BCL2*), Fos Proto-Oncogene, AP-1 transcription factor subunit (*FOS*), G protein subunit alpha O1 (*GNAO1*), and phospholipase C beta 1 (*PLCB1*) were identified. In Figure 4, the top 10 enriched KEGG pathways are illustrated.

PPI Network Construction: Using STRING, we constructed the PPI network of significant overlapped genes associated with HER2-targeted drug resistance illustrated in Figure 4. The PPI network revealed BCL2 as a central hub, with connections to *FOS*, C-X-C motif chemokine receptor 4 (*CXCR4*), C-X-C motif chemokine ligand 10 (*CXCL10*), interleukin 6 receptor (*IL6R*), interleukin 6 cytokine family signal transducer (*IL6ST*), insulin receptor substrate 2 (*IRS2*), death-associated protein kinase 1 (*DAPK1*), and cadherin 2 (*CDH2*). Separate clusters involving *PLCB1*, adenylate cyclase 1 (*ADCY1*), and *GNAO1* were also identified. Additional central nodes in the network, such as *ANK2,* ankyrin 3 (*ANK3*), and *CDH2*, were observed, hinting at the potential roles in cell adhesion and signaling.

By employing an integrated approach, we identified a set of strong candidate genes potentially involved in HER2 drug resistance in breast cancer. These hub genes (*BCL2*, *FOS*, *CXCR4*, *CXCL10*, *PLCB1*, *ADCY1*, and *GNAO1*) were part of interconnected pathways and were often central in the PPI network, suggesting critical roles in underlying their biological mechanisms of HER2-targeted drug resistance, and deserve further validation. Detailed information on the interaction between miRNAs and potential hub genes is shown in Supplementary Table S1.

### 3.5. Survival Analysis and Immunohistochemistry of Hub Genes

**Figure 5:**
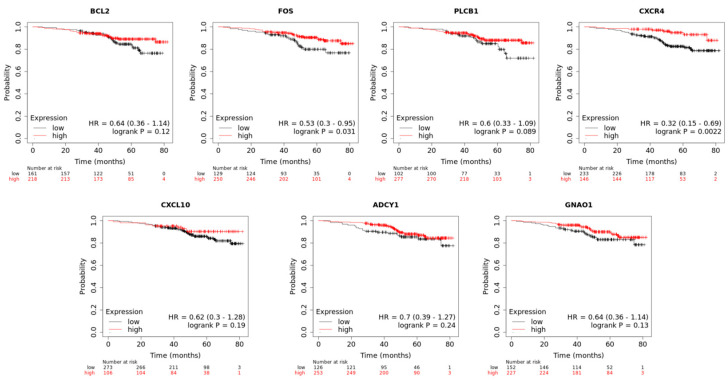
Kaplan–Meier survival plots for hub genes in HER2+ breast cancer. The plots illustrate the overall survival associated with the expression levels of hub genes BCL2, FOS, CXCR4, CXCL10, PLCB1, ADCY1, and GNAO1. Statistically significant associations were found for CXCR4 and FOS, indicating longer overall survival in patients with high expression of these genes. The hazard ratios (HRs) and 95% confidence intervals (CIs) are provided in the plot for each gene.

Survival analysis: We utilized Kaplan–Meier plots to evaluate the impact of hub genes on overall survival in HER2+ breast cancer. Specifically, the high expression of *CXCR4* was correlated with longer overall survival, with a hazard ratio (HR) of 0.32 (95% CI 0.15–0.69) and a log-rank *p*-value of 0.0022. In addition, *FOS* also exhibited an association with favorable survival outcomes (HR = 0.53, 95% CI 0.3–0.95; *p* = 0.0311). Other analyzed genes, including *BCL2*, *CXCL10*, *PLCB1*, *ADCY1*, and *GNAO1*, did not demonstrate statistically significant associations with overall survival at the *p* < 0.05 threshold, although trends were observed, such as in PLCB1 with an HR of 0.6 (95% CI 0.33–1.09; *p* = 0.089). The results of the survival analysis are shown in Figure 5.

**Figure 6:**
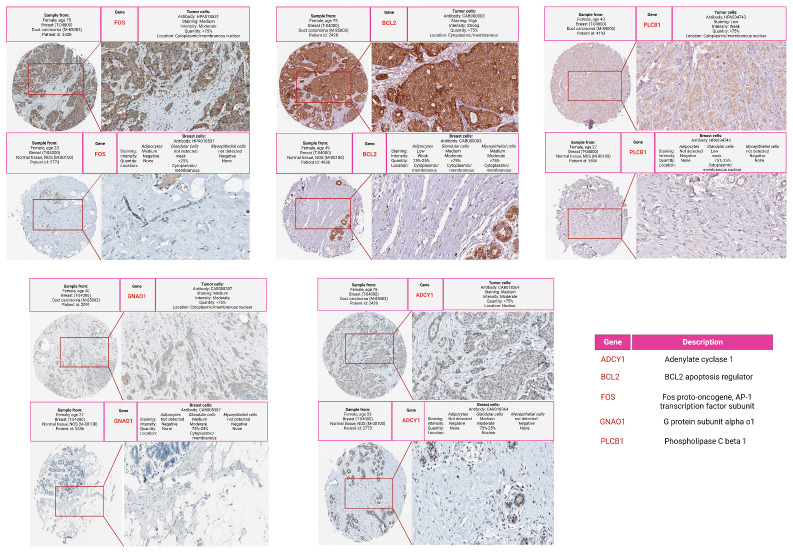
Immunohistochemical (IHC) staining comparison of hub genes in normal and tumor breast tissue. The figure illustrates the differences in staining intensity and patterns for genes including FOS, BCL2, PLCB1, ADCY1, and GNAO1. A noticeable increase in staining in tumor cells for the FOS gene emphasizes its enhanced expression in cancerous tissues, while other genes demonstrate marked or subtle changes. The data for CXCR4 and CXCL10 were not available from the Human Protein Atlas.

IHC findings: We employed the results from immunohistochemistry (IHC), sourced from the Human Protein Atlas, to assess the expression patterns of hub genes in normal and tumor breast tissue. A general increase in staining and intensity was observed in tumor cells for various hub genes, suggesting the intricate cellular modifications that might be contributing to tumorigenesis. In particular, the FOS gene demonstrated a noticeable increase in staining in tumor cells, indicating enhanced expression in the cancerous tissues. Other genes, such as *BCL2*, revealed marked differences in staining, while *PLCB1*, *ADCY1*, and *GNAO1* showed more subtle changes. The data for *CXCR4* and *CXCL10* were not available from the Human Protein Atlas. The IHC comparison result between normal and tumor samples for the hub genes is illustrated in Figure 6. Detailed information on miRNAs regulating these potential hub genes retrieved from miRTarBase can be found in Supplementary Table S2.

## 4. Discussion

HER2+ breast cancer represents a substantial portion of all breast cancer cases, yet limited biomarkers are available to predict responses to HER2-targeted therapies. Despite their potential to enhance treatment efficiency in personalized medicine, few studies have explored miRNA biomarkers for the HER2+ breast cancer drug response. Standard techniques used in cancer biomarker studies are microarray analysis and data mining [41]. In HER2-breast cancer drug resistance studies, the minimal overlap observed in identified miRNA panels reflects the complex biology of regulator miRNA expression in HER+ breast cancer. Therefore, while valuable, a single microarray dataset might not be able to capture the entire landscape of this complexity.

Our study tackled this challenge by adopting a comprehensive meta-analysis of multiple miRNA datasets. We systematically identified and validated significant miRNAs associated with HER2 drug resistance by integrating data across platforms, testing for heterogeneity, and applying a random-effect model. We identified significant miRNAs associated with HER2 drug resistance in breast cancer and predicted their target genes. By manipulating three external mRNA datasets, we validated those target genes and further explored the biological significance of these genes with GO and KEGG enrichment analysis. Next, we constructed a PPI network to identify the most significant hub genes associated with HER2 drug resistance. These genes serve as potential key regulators, connecting various biological functions and pathways. Furthermore, their prognostic value and clinical relevance were examined through survival analysis and IHC results.

CXCR4 has been identified as a key regulator of metastasis in HER2 breast cancer, with studies indicating that its inhibition can have a favorable impact on prognosis [42,43,44]. Interestingly, our analysis has revealed an association between higher *CXCR4* expression and improved overall survival in HER2 breast cancer patients. This discrepancy may suggest a more complex interaction within the tumor microenvironment, where *CXCR4*’s impact on survival could be modulated by other factors, including miRNAs and interactions with other signaling pathways. Furthermore, Lefort et al. [43] showed the potential benefit in reducing tumor growth and metastasis, especially in trastuzumab-sensitive and trastuzumab-resistant HER2 breast cancers, by targeting the CXCR4/CXCL12 axis. Moreover, *CXCR4*’s involvement in drug resistance, particularly in trastuzumab treatment, has been well documented, reinforcing its critical role in the therapeutic landscape of HER2 breast cancer [45,46]. The complexity of *CXCR4*’s role extends to its regulation by miRNAs. Validation studies have identified miR-139 as a direct regulator of *CXCR4* in various cancers, including breast cancer [47,48,49]. Our research further supports this association, highlighting that miR-139-5p modulates *CXCR4* in HER2 breast cancer drug resistance.

The downregulation of the *FOS* gene has been identified in aggressive breast cancer subtypes, including basal, HER2-positive, and luminal B cases, with decreased expression levels correlating with advanced cancer stages [50]. Our study further substantiates previous findings regarding the positive correlation between *FOS* expression and overall survival (OS) in breast cancer [50] and specifically highlights an association between increased *FOS* expression and improved survival outcomes in HER2 breast cancer patients. Moreover, we have identified *FOS* as a target of hsa-miR-5586-5p. While the role of hsa-miR-5586-5p in breast cancer remains largely unexplored, existing research has documented its significance in modulating chemotherapy response in squamous cell carcinomas (SCCs) [51]. Thus, understanding the molecular mechanisms by which hsa-miR-5586-5p may regulate *FOS* expression represents a critical area for further research, with potential implications for developing targeted therapeutic strategies in HER2+ breast cancer.

Additionally, our study explored the roles of other hub genes, including *BCL2*, *PLCB1*, *ADCY1*, and *GNAO1*, within the HER2-positive breast cancer context. Though these genes did not exhibit statistically significant associations in survival analysis, their inclusion in the PPI network and differences in staining patterns observed through immunohistochemical (IHC) staining underscore their potential relevance. The *BCL2* gene, while previously known for its extensive study in breast cancer, especially ER-positive breast cancer [52,53], and its subtype-specific prognostic role [54], exhibited a marked change in its staining pattern within HER2-positive breast cancer according to the IHC result. In this specific context, BCL2 has been identified as a potential therapeutic target to restore sensitivity to T-DM1 [55]. We have identified BCL2 as the target of hsa-miR-96-5p. Given that hsa-miR-96 is known as a candidate diagnostic marker in breast cancer [56], its potential role in regulating *BCL2* could be significant in drug resistance within HER2-positive breast cancer.

Besides *CXCR4*, our study found that hsa-miR-139-5p also regulates *GNAO1, CXCL10*, and *ADCY1*. In the context of HER2-positive breast cancer, *CXCL10* emerges as being particularly significant, implicated in trastuzumab responses through interferon-gamma-inducible protein 16 (IFI16)-dependent STING signaling. The under-expression of this pathway, modulated by histone transferase EZH2, leads to suppressed CXCL10/11 expression and thereby contributes to trastuzumab resistance [57]. This intricate regulation by both histone transferase and miRNA exemplifies the complexity of epigenetic mechanisms governing HER2 breast cancer.

While there is evidence for the potential of enhancing chemotherapy efficacy through *ADCY1* modulation in breast cancer [58], and a correlation has been established between overexpressed *GNAO1* and poor prognosis in gastric cancer [59], there is a conspicuous lack of studies focusing on the role of these genes in HER2-positive breast cancer drug resistance. Therefore, further investigation into these genes, as well as the miRNA-mediated modulation mechanisms that may contribute to resistance to targeted drugs, is necessary.

This study has some limitations. Primarily, the analytical miRNA data were sourced from public databases, some of which contain limited sample sizes. This constraint potentially affects the robustness of our findings related to miRNA associated with HER2 drug resistance. To enhance reliability and confidence in our results, we complemented the analysis by utilizing external mRNA datasets to validate the target genes of the significant miRNAs. Future investigations employing larger, well-curated sample sizes could provide more definitive insights. In addition, further experimental validation of hub genes identified in this study can elucidate the complex epigenetic mechanisms governing HER2 breast cancer drug resistance and further validate our findings.

## 5. Conclusions

In this study, we utilized bioinformatics and meta-analysis to identify miRNAs, such as miR-139-5p, miR-96-5p, and miR-5586-5p, associated with HER2-targeted drug resistance in breast cancer. By addressing the issue of non-overlapping miRNA panels across studies and validating our findings using external mRNA datasets, we have provided preliminary insights into the molecular mechanisms underlying HER2-targeted drug resistance. These insights may have implications for the development of targeted therapeutic strategies for patients with HER2+ breast cancer. Although our study lays important groundwork, further experimental validation is required to fully understand these complex interactions. Our results reinforce the significant role of miRNAs in shaping the therapeutic landscape of HER2-positive breast cancer.

## Figures and Tables

**Table 1:** Characteristics of the included datasets.

	GSE Accession No.	No. of Samples	Platform	Description	Country	PMID	URL and Access Date
miRNA	GSE47011	6	miRCURY LNA microRNA Array	Screening for microRNAs involved in the development of trastuzumab resistance using SKBR3 cells	China	24615544	https://www.ncbi.nlm.nih.gov/geo/query/acc.cgi?acc=GSE47011 accessed on 22 May 2023
	GSE197822	12	Affymetrix Multispecies miRNA-4 Array	Determining the differences in miRNA expression between breast cancer cell lines (SKBR3 and BT474) and their resistant pairs.	Spain	Not available	https://www.ncbi.nlm.nih.gov/geo/query/acc.cgi?acc=GSE197822 accessed on 22 May 2023
	GSE101841	103	Affymetrix GeneChip miRNA 4.0 Array	Screening of serum-based miRNA signature of patients resistant to trastuzumab	China	29691399	https://www.ncbi.nlm.nih.gov/geo/query/acc.cgi?acc=GSE101841 accessed on 22 May 2023
mRNA	GSE132055	15	Illumina HiSeq 2000	RNA-seq of three HER2+ breast cancer anti-HER2-resistant cell models	USA	31420371	https://www.ncbi.nlm.nih.gov/geo/query/acc.cgi?acc=GSE132055 accessed on 22 May 2023
	GSE89216	8	Affymetrix Human Gene 2.0 ST Array	Identify acquired resistance mechanisms to anti-HER2 antibodies trastuzumab and pertuzumab, and to the combined trastuzumab/pertuzumab or pertuzumab/T-DM1 therapy	Spain	Not available	https://www.ncbi.nlm.nih.gov/geo/query/acc.cgi?acc=GSE89216 accessed on 22 May 2023
	GSE121105	21	Illumina HiSeq 2000	RNA sequencing of BT474 cells treated with trastuzumab or trastuzumab + pertuzumab and BT474-derived cells resistant to trastuzumab or trastuzumab + pertuzumab	USA	31690671	https://www.ncbi.nlm.nih.gov/geo/query/acc.cgi?acc=GSE121105 axxessed on 22 May 2023

## Data Availability

All datasets used in this study can be found in the Gene Expression Omnibus database at https://www.ncbi.nlm.nih.gov/geo/. (accessed on 22 May 2023).

## Supplementary Materials

The following supporting information can be downloaded at https://www.mdpi.com/article/10.3390/cancers16233962/s1: Figure S1: Selection method; Figure S2: Exploratory analyses for miRNA datasets GSE47011, GSE197822, and GSE101841 using GEO2R; Table S1: Interaction between miRNAs and potential hub genes; Table S2: Details on miRNAs regulating identified potential hub genes. References [47,48,49,51,56,57,60,61,62] are cited in the supplementary materials.

## Abbreviations

ADCY1	Adenylate Cyclase 1
BCL2	B-cell lymphoma 2
CI	Confidence Interval
CXCL10	C-X-C Motif Chemokine Ligand 10
CXCR4	C-X-C Chemokine Receptor Type 4
ER	Estrogen Receptor
EZH2	Enhancer of Zeste Homolog 2
FOS	Fos Proto-Oncogene
GNAO1	Guanine Nucleotide Binding Protein Alpha O Subunit
GO	Gene Ontology
HER2	Human Epidermal Growth Factor Receptor 2
HR	Hazard Ratio
IFI16	Interferon-Induced Protein 16
IHC	Immunohistochemistry
KEGG	Kyoto Encyclopedia of Genes and Genomes
miRNA	MicroRNA
OS	Overall Survival
PLCB1	Phospholipase C Beta 1
PPI	Protein–Protein Interaction
SCCs	Squamous Cell Carcinomas
STING	Stimulator of Interferon Genes
T-DM1	Trastuzumab Emtansine
