# Supplementary material for: Mechanism of miRNAs and miRNA-mRNA Regulatory Networks in Modulating Drug Resistance in HER2-Positive Breast Cancer: An Integrative Bioinformatics Approach
Source: Cancers (Basel). 2024 Nov 26;16(23):3962. doi: 10.3390/cancers16233962 (PMC11640410; doi:10.3390/cancers16233962)
Supplement: Supplementary file 1 [file cancers-16-03962-s001.zip › cancers-3296004-supplementary.pdf]

Supplementary files:

Supplementary figure:

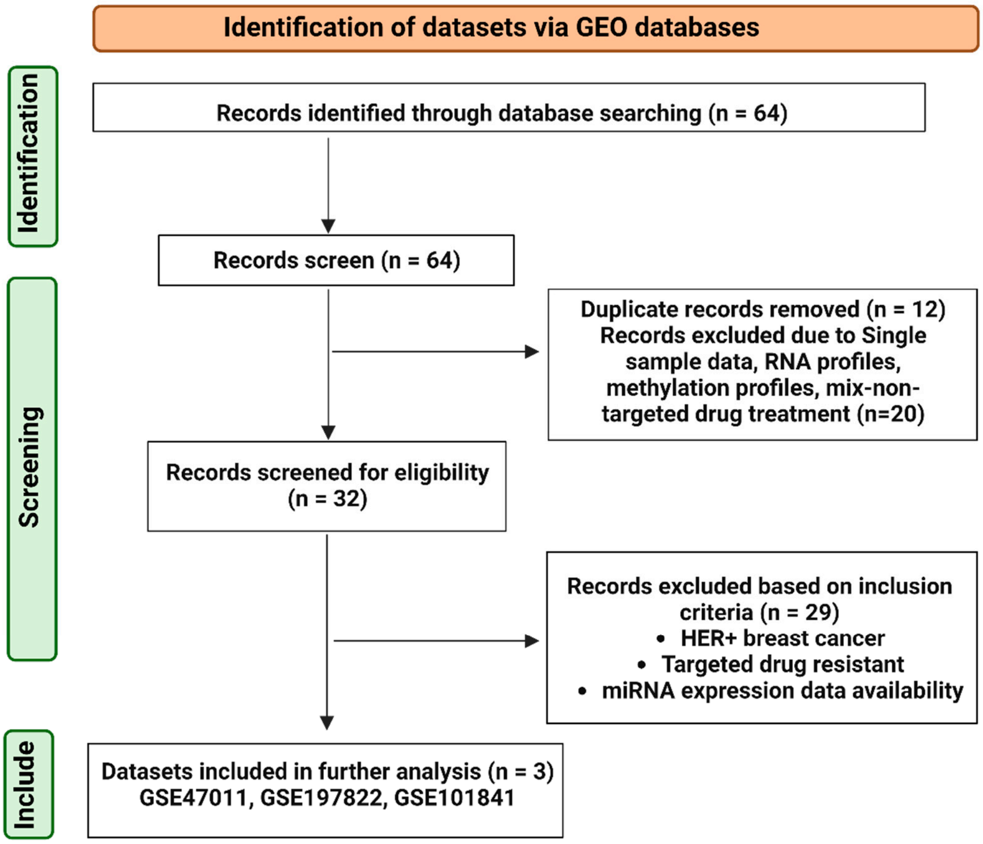

Figure S1: Selection method

PRISMA diagram of our dataset search strategy

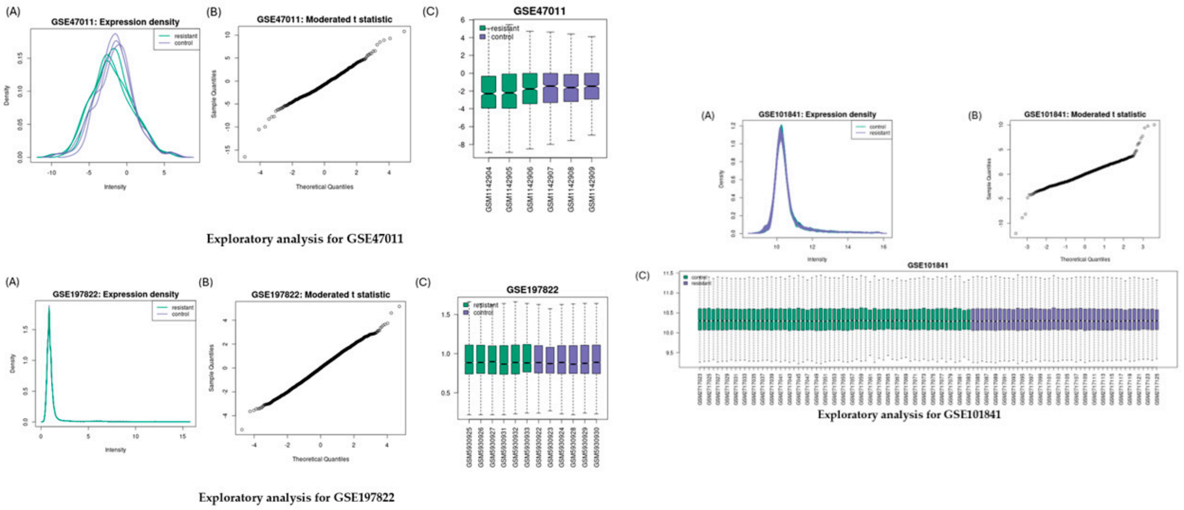

Exploratory analyses for miRNA datasets

Figure S2: Exploratory analyses for miRNA datasets GSE47011, GSE197822, and GSE101841 using GEO2R. *GSE47011 (Panels A–C): (A) Expression Density Plot: Density plot showing the distribution of expression intensity values for resistant and control samples in GSE47011, demonstrating consistent expression distributions across groups. (B) Moderated t-Statistic Q-Q Plot: Q-Q plot for the moderated t-statistics in GSE47011. The close alignment of points along the diagonal line indicates that the assumptions for differential expression analysis are met. (C) Box Plot of Expression Levels: Box plot showing the expression level distributions across resistant and control samples in GSE47011, confirming uniform expression across samples and supporting data normalization. GSE197822 (Panels A–C): (A) Expression Density Plot: Density plot showing expression intensity distributions for resistant and control samples in GSE197822, indicating a similar distribution pattern across groups. (B) Moderated t-Statistic Q-Q Plot: Q-Q plot for the moderated t-statistics in GSE197822, showing good alignment along the diagonal, confirming that statistical assumptions for differential expression analysis hold. (C) Box Plot of*

Expression Levels: Box plot displaying expression level distributions for resistant and control samples in GSE197822, with similar distribution across samples. GSE101841 (Panels A–C): (A) Expression Density Plot: Density plot for GSE101841 showing the distribution of expression intensities for resistant and control samples, with both groups exhibiting similar overall patterns. (B) Moderated t-Statistic Q-Q Plot: Q-Q plot for the moderated t-statistics in GSE101841, showing alignment with the diagonal line, supporting the appropriateness of the statistical assumptions. (C) Box Plot of Expression Levels: Box plot showing expression level distributions across resistant and control samples in GSE101841, indicating consistent expression across samples and groups.

Supplementary tables:

miRNA-mRNA interaction information

**Hub genes**	**BCL2**	**FOS**	**CXCR4**	**CXCL10**	**PLCB1**	**ADCY1**	**GNAO1**
**Regulators**	hsa-miR-96-5p	hsa-miR-5586-5p	hsa-miR-139-5p	hsa-miR-139-5p	hsa-miR-139-5p	hsa-miR-139-5p	hsa-miR-139-5p
	hsa-miR-139-5p	hsa-miR-139-5p					

Supplementary Table S1: Interaction between miRNAs and potential hub genes

Information on key miRNAs from miRTarBase and miRBase

Hairpin miRNA	Chromosome	mature miRNA	target gene	miRBase ID	sequence	Evidence	Exp	DRVs in miRNA	SNPs in miRNA	Target Gene Pathway Associations	Ref.
hsa-miR-96	chr7: 129774692-129774769	hsa-miR-96-5p	BCL2	MIRT027929	9| UUUGGCACUAGCACAUUUUUGCU |31	Experimental	Cloned	2	7	Apoptosis, cell survival	[1, 2]
hsa-miR-139	chr11: 72615063-72615130	hsa-miR-139-5p	BCL2	MIRT732232	7| UCUACAGUGCACGUGUCUCCAGU |29	Experimental	Cloned	2	7	Immune modulation, cell growth	[3, 4]
hsa-miR-139	chr11: 72615063-72615130	hsa-miR-139-5p	FOS	MIRT006976	7| UCUACAGUGCACGUGUCUCCAGU |29	Experimental	Cloned	2	7	Immune modulation, cell growth	[4, 5]
hsa-miR-5586	chr14: 59646962-59647020	hsa-miR-5586-5p	FOS	MIRT476772	1| UAUCCAGCUUGUUACUAUAUGC |22	Not_experimental	NB	NB	7	Cancer pathways related to drug response	[6, 7]

hsa-miR-139	chr11:72615063-72615130	hsa-miR-139-5p	CXCR4	MIRT054160	7| UCUACAGUGCACGUGUCUCCAGU |29	Experimental	Cloned	2	7	STING signaling, immune response pathways	[5, 8, 9]

Supplementary Table S2: Details on *miRNAs regulating identified potential hub-genes*

Information on hairpin sequence and chromosome is retrieved from miRbase, the rest is retrieved from MiRTarBase.

1. Hafner, M., et al., *Transcriptome-wide identification of RNA-binding protein and microRNA target sites by PAR-CLIP.* Cell, 2010. **141**(1): p. 129-41.
2. Xiong, D.-d., et al., *A nine-miRNA signature as a potential diagnostic marker for breast carcinoma: An integrated study of 1,110 cases.* Oncology reports, 2017. **37 6**: p. 3297-3304.
3. Li, Q., et al., *miR-139-5p Inhibits the Epithelial-Mesenchymal Transition and Enhances the Chemotherapeutic Sensitivity of Colorectal Cancer Cells by Downregulating BCL2.* Sci Rep, 2016. **6**: p. 27157.
4. Cheng, C.W., et al., *MiR-139 Modulates Cancer Stem Cell Function of Human Breast Cancer through Targeting CXCR4.* Cancers (Basel), 2021. **13**(11).
5. Bao, W., et al., *HER2 interacts with CD44 to up-regulate CXCR4 via epigenetic silencing of microRNA-139 in gastric cancer cells.* Gastroenterology, 2011. **141**(6): p. 2076-2087.e6.
6. Whisnant, A.W., et al., *In-depth analysis of the interaction of HIV-1 with cellular microRNA biogenesis and effector mechanisms.* mBio, 2013. **4**(2): p. e000193.
7. Fekete, J.T., Á. Welker, and B. Győrffy, *miRNA Expression Signatures of Therapy Response in Squamous Cell Carcinomas.* Cancers (Basel), 2020. **13**(1).
8. Luo, H.N., et al., *MiR-139 targets CXCR4 and inhibits the proliferation and metastasis of laryngeal squamous carcinoma cells.* Med Oncol, 2014. **31**(1): p. 789.
9. Ong, L.T., et al., *IFI16-dependent STING signaling is a crucial regulator of anti-HER2 immune response in HER2+ breast cancer.* Proc Natl Acad Sci U S A, 2022. **119**(31): p. e2201376119.
